# Effect of finishing protocols and staining solutions on color stability of dental resin composites

**DOI:** 10.1002/cre2.555

**Published:** 2022-03-29

**Authors:** Chamunorwa Marufu, Bernina K. Kisumbi, Olivia A. Osiro, Fred O. Otieno

**Affiliations:** ^1^ Department of Conservative and Prosthetic Dentistry, School of Dental Sciences University of Nairobi Nairobi Kenya

**Keywords:** composite materials, esthetic dentistry, restorative dentistry, surface chemistry/properties

## Abstract

**Objective:**

The objective of this study was to evaluate the effect of finishing protocol and exposure to staining solutions on color stability of dental resin composites.

**Materials and Methods:**

A nanofill and microhybrid composite, three finishing protocols (mylar, *Soflex* disc, and white polishing stone) and four staining solutions (tea, red wine, khat extract—two concentrations, control—distilled water) were evaluated. A digital spectrophotometer was used for color change (Δ*E*) measurements employing the CIE‐Lab‐color system. Paired/independent‐sample *t* test and two‐way analysis of variance (ANOVA) followed by Tukey's honestly significant difference posthoc test were used for inferential statistics at *α* = .05.

**Results:**

*Soflex* finish was associated with least staining and comparable color stability for the two materials in tea and red wine. In Khat 2, microhybrid composite had statistically significant better color stability than nanofill for *Soflex* finish (14 days *t* = 3.270, *p* = .011). For microhybrid composite, mylar resulted in highest mean Δ*E*, whereas *Soflex* recorded the least in all staining solutions. For nanofill composite, white stone resulted in highest mean Δ*E*, whereas *Soflex* demonstrated the least mean Δ*E* in all staining solutions, except red wine where mylar demonstrated the least mean Δ*E*. For mylar finish, nanofill demonstrated statistically significant better color stability than microhybrid in both red wine (14 days *t* = 4.902, *p* = .001) and Khat 1 (14 days *t* = 3.252, *p* = .012). For stone finish, microhybrid demonstrated statistically significant better color stability than nanofill in all staining solutions (14 days *t* ≥ 4.785, *p* ≤ .001). Two‐way ANOVA showed a statistically significant difference in mean Δ*E* between and within specimens (*F* = 42.658, *p* < .001). All staining solutions caused clinically unacceptable discoloration for mylar and white stone finish. For *Soflex* finish, red wine produced clinically unacceptable color difference beyond 48 h.

**Conclusion:**

There was a difference in color stability of resin composites depending on filler type, further influenced by finishing protocol. *Soflex* disc finish results in better color stability than mylar and white stone in both microhybrid and nanofill composites.

**Clinical significance:**

Esthetic dental restorations such as resin composites are routine in contemporary restorative practice. Color stability of composites may be influenced by surface finish, dependent on the filler type, and consumption of chromogenic substances such as khat. To prolong their service, selection of suitable finishing protocols is an important consideration.

## 1 INTRODUCTION

Dental resin composites are ceramic‐reinforced polymer systems applied in direct and indirect tooth‐colored restorations (Osiro et al., 2016). Since their introduction in the 1960s, they have become increasingly popular for both anterior and posterior restorations (Demarco et al., 2017; Sarkis, 2012). This is due to growing esthetic demands from patients and a paradigm shift in philosophies of operative dentistry that have resulted in transition from GV Blacks' principles of “extension for prevention” to current concepts of minimal intervention dentistry (Osiro et al., 2019).

Direct composite restorations have several advantages as follows: they are less technically demanding, cheaper than indirect restorations, do not cause wear to opposing dentition, and can be easily repaired (Kohli & Bhatia, 2015). Moreover, bonding mediated by dental adhesive systems favor conservative preparations and light‐activated versions enable operator‐controlled working time (Ritter et al., 2017). However, their disadvantages include polymerization shrinkage stress resulting in microleakage, color instability, incomplete polymerization, and limited curing depth (Ritter et al., 2017). Nonetheless, current advances have attempted to address the inhomogeneous polymerization and limited depth of cure by employing strategies such as nanofiller technology, silorane instead of methacrylate resins and photoinitiators other than camphoroquinone (Gonçalves, 2018). Resin composites have an average clinical longevity of 6–10 years (Henry, 2009).

The failure behavior of composites differs in the anterior and posterior regions. Posterior composites fail primarily due to secondary caries and fracture (Opdam et al., 2014), whereas anterior composites commonly fail due to esthetic factors such as color alterations (Berber et al., 2013; Catelan et al., 2013; Fernando Demarco et al., 2015). Discoloration of resin composites may be intrinsic or extrinsic (Singh, 2014). Intrinsic discolouration is permanent (Malekipour et al., 2012) and is related to the materials' composition, that is, the matrix, filler type and amount, photoinitiator system, and percentage of remaining double carbon bonds as expounded by the degree of conversion (Catelan et al., 2013). On the other hand, extrinsic discoloration may be due to accumulation of plaque biofilm and related staining, low degree of polymerization, exposure to environmental factors including heat, water, food colorants, and ambient and ultraviolet light. Notably, color change is often a result of a combination of these factors. Finishing and polishing protocols of composites are also an important determinant of extrinsic discoloration (Ashok & Jayalakshmi, 2017).

Finishing removes residual surface imperfections following contouring by cutting or grinding, whereas polishing provides lustre on a material surface. This is essential for maintenance of oral health, function, and esthetics (Kumari et al., 2015). Finishing and polishing minimize extrinsic discoloration of restorations by preventing accumulation of plaque biofilm and staining agents (Samra et al., 2008; Schmitt et al., 2011). Typically, the technique employs a stepwise approach with methodical gradual use of finer instruments including diamond and carbide‐finishing burs, abrasive‐impregnated rigid points, impregnated rubber cups and points, aluminum oxide‐coated abrasive discs, abrasive strips, and polishing pastes (Kumari et al., 2015). The aim is first to contour the restoration using diamond burs, carbide burs, or coarse abrasive‐coated discs. This is followed by finishing with either fine or extra‐fine diamond burs, carbide burs, white aluminum oxide stones, white Arkansas stones, or medium and fine abrasive coated discs. The final step is polishing to achieve an enamel‐like lustre using fine and extra‐fine polishing paste (aluminum oxide or diamond), abrasive coated discs, silicon carbide‐impregnated brushes or diamond‐impregnated rubber polishing discs, cups, or points. It is recommended that all the finishing and polishing instruments within a selected system are used in the proper sequence (Patel et al., 2016). Early studies on finishing of resin‐based restorations showed that very smooth surfaces could be obtained when restorations were allowed to set in contact with mylar strips (Heath & Wilson, 1976). However, composites polymerized in contact with a mylar strip leave a resin‐rich surface layer, the oxygen inhibition layer, which is easily abraded in the oral environment exposing rough inorganic fillers, tends to absorb more water and is more prone to staining (Schmitt et al., 2011; Uçtaşli et al., 2007). There is a tendency to consider the smooth surface achieved with the mylar strip a final finish (Patel et al., 2016), but it is associated with more color change than the *Soflex* disc finish (Kumari et al., 2015; Yew et al., 2013).

Several staining agents and their effects on color stability of resin composites have been evaluated, including red wine, tea, and khat. Red wine has been associated with greater color change comparatively (Catelan et al., 2013; Kisumbi, 1998). The effect of tea may be worse when sugar is added (Guler et al., 2005) or chlorhexidine rinse is used concomitantly (Omata et al., 2006). Khat is considered a drug of abuse with less addictive potential than alcohol and tobacco (Aden et al., 2006), and its use has been linked to a range of health issues affecting the gastrointestinal, cardiovascular, and reproductive systems (Al‐Motarreb et al., 2002). Oral conditions associated with khat include periodontal disease, dry mouth, dental caries, oral mucosal lesions, temporomandibular joint disorders, and oral cancer (Astatkie et al., 2015). Staining of teeth from khat chewing is caused by tannins together with the presence of khat‐favoring chromogenic bacteria (Aden et al., 2006; Al‐Maweri et al., 2018).

The influence of the filler type on finishing is an important variable when studying the effect of staining solutions and finishing protocols on the color stability of resin composites (Schmitt et al., 2011). Further, information is scarce on the staining effects of khat, yet it is consumed in large quantities by certain communities in Africa, Asia, and Europe (Aden et al., 2006; Al‐Maweri et al., 2018; Al‐Motarreb et al., 2002; Astatkie et al., 2015). Therefore, the aim of this study was to evaluate the effect of three finishing protocols and exposure to four staining solutions on the color stability of dental resin composites, testing the hypothesis that there is no difference in the color stability of microhybrid and nanofill composites.

## 2 MATERIALS AND METHODS

### 2.1 Materials and sample size

Two dental resin composites were evaluated as follows: a nanofill, *Filtek Z350* (3M ESPE, St Paul, MN, USA), and a microhybrid, *Vit‐l‐escence* (Ultradent, Inc., South Jordan, UT, USA). The materials were subjected to three finishing and polishing protocols, namely Mylar strip (Maquira Industries, Brazil), *Soflex* polishing discs (3M ESPE Dental Products, Neuss, Germany), and White polishing stone (Prima Dental, Gloucester, UK) in four staining solutions, tea, red wine, and khat extract (diluted to two different concentrations), whereas distilled water was the control. Color stability was determined as specified in ISO 7496:2000 for color stability of dental materials, with five test specimens for each finishing protocol and staining solution for both materials. Thus, for each material, there were three finishing protocols subjected to four staining solutions and one control. Each group contained five samples, resulting in a total of 150 samples as summarized in Table 1.

**Table 1:** Summary of sample size distribution

Material	Polishing protocol	Khat (K1)	Khat (K2)	Tea (T)	Red wine (R)	Distilled water (C)	No. of specimens
*Filtek Z350 XT*	Mylar strip (M)	5	5	5	5	5	25
	*Soflex* disc (D)	5	5	5	5	5	25
	White stone (S)	5	5	5	5	5	25
*Vit‐l‐scence*	Mylar strip (M)	5	5	5	5	5	25
	*Soflex* disc (D)	5	5	5	5	5	25
	White stone (S)	5	5	5	5	5	25
Total		30	30	30	30	30	150

#### 2.1.1 Specimen preparation

Specimen discs were prepared (*n* = 150, 75 *Vit‐l‐escence* and 75 *Filtek Z350 XT*) in perspex moulds 8 mm diameter × 2 mm depth. A single layer of composite was packed into a mould and light cured on both sides for 40s (Blueluxcer M385, Monitex Industrial Co. Ltd, Taiwan), including a segment of dental floss to suspend the specimens in the staining solutions and fasten a label. Before curing, the irradiance intensity of the unit was checked using a handheld analog radiometer (Apoza, New Taipei City, Taiwan) and determined to be 1000 mW/cm^2^.

#### 2.1.2 Finishing/polishing protocols

Mylar finish (M) was achieved by curing with Mylar strips on both sides of the specimen while the other protocols had a Mylar finish on one side and the other side exposed. The exposed side was polished using either a *Soflex* disc or white stone. Coarse, medium, fine, and superfine grit sequence of *Soflex* discs (D) held on a slow‐speed hand piece were used sequentially for 30 s each. After each polishing, the specimens were rinsed and air‐dried for 10 s before the next step. White polishing stones (S) were used for 2 min on a slow‐speed hand piece with minimal pressure. After treatment, all the specimens were placed in distilled water at room temperature for 24 h, to allow for rehydration and completion of polymerization (Afzali et al., 2015; Mundim et al., 2010), after which baseline color measurements were recorded.

#### 2.1.3 Staining protocols

Red wine (Robertson Winery, South Africa) (R), alcohol content of 7.5% and pH 3.4 was used as is. Black tea (Ketepa, Kenya) (T), pH 5.5, was brewed by placing a tea bag in 250 ml of boiling water for 4 min with no sugar added and then allowed to cool. The khat extraction protocol was previously described (Dimba et al., 2003) and is a modification of the methanolic extraction protocol (Lee, 1995), excluding the alkaloid purification to minimize acid or basic residues in the extract. Briefly, 870 g of fresh khat shots were chopped into small pieces and covered in methanol, the mixture agitated in a sonicator at room temperature, and filtered through an 11 µm filter to produce 10 g of extract. The green methanolic extract was then placed into a rotorvapor vacuum drier at 337 mbar until all the methanol evaporated, then refrigerated at 4°C awaiting testing. A dilution factor of 87 was derived from the extraction ratio and used to prepare the first Khat solution (K1). To mimic the effect of saliva when the khat plant is being chewed, a second solution of Khat (K2) was made at 1:3 dilution of K1. Specimens were suspended in 10 ml of the solutions maintained at 37°C in light‐proof containers for 2 weeks. Test solutions were refreshed at the end of the first week.

### 2.2 Color measurements

A digital spectrophotometer (Vita Easyshade, Vita Zahnfabrik) based on the CIE‐Lab‐color (Commission International de l' Eclairage L*a*b color) system was used for all color measurements against a white background. Color measurements were taken after 6 h and 1, 2, 4, 7, 10, and 14 days. The mean value for the five specimens was calculated. Paired‐sample and independent‐sample *t* test was used to determine the mean color difference at baseline and at the end of staining between the two materials, whereas two‐way analysis of variance (ANOVA) was used to determine the mean color difference within or between groups of finishing protocols and staining solutions followed by Tukey's honestly significant difference posthoc test at 95% confidence level.

## 3 RESULTS

At the end of the staining period all specimens, except (D) finished micro‐hybrid specimens (*Vit‐l‐escence*) in the K1 and K2 staining solutions, demonstrated a clinically unacceptable color difference (Δ*E*). Total Δ*E* above 2.6 units is considered clinically perceptible, whereas Δ*E* of 5.5 is considered clinically unacceptable, represented in the figures by the black and white arrows, respectively.

**Figure 1:**
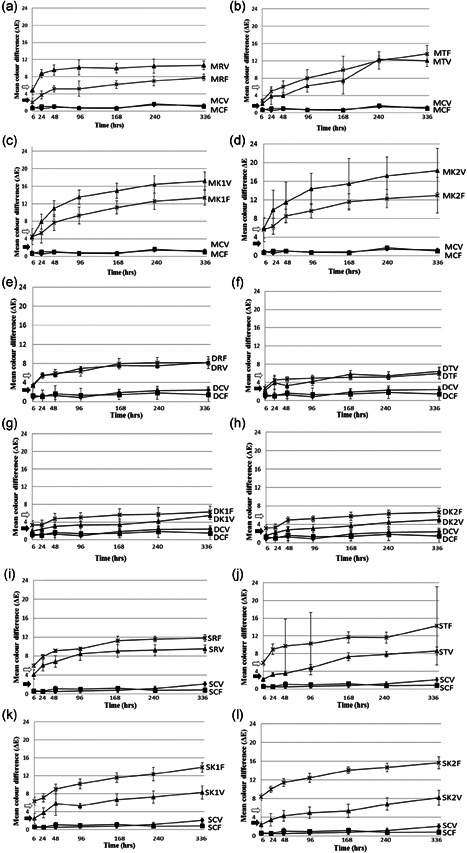
Mean color difference (Δ*E*) for Mylar (M), Soflex disc (D) and White stone (S) finished specimens of *Vit‐l‐escence* (V) and *Filtek* (F) in red wine (R)—(a), (e), and (i); tea (T)—(b), (f), and (j); Khat 1 (K1)—(c), (g), and (k); and, Khat 2 (K2)—(d), (h), and (l). Control (C); black arrow, clinically perceptible Δ*E* level; white arrow, clinically unacceptable Δ*E* level (error bars denote SD)

Figure 1 compares the mean Δ*E* of *Vit‐l‐escence* and *Filtek* samples in comparison with the controls: Mylar finish (A, B, C, and D), Soflex disc finish (E, F, G, and H), White stone finish (I, J, K, and L) in red wine (A, E, and I), tea (B, F, and J), Khat 1 (C, G, and K) and Khat 2 (D, H, and L). Table 2 shows the results of the paired‐sample *t* tests for the same.

**Table 2:** Paired‐sample *t* tests of mean Δ*E* of M, *Soflex* D, and S finished V and F specimens in R, T, K1, and K2 at baseline and at 14 days

Specimen	*n*	*M*		95% Confidence interval		*df*		
			SD	Lower bound	Upper bound		*t* test	*p*
MRV	5	10.66	1.02	1.50	4.57	8	4.902**	.001
MRF	5	7.82	0.79					
MTV	5	12.00	1.27	−3.99	0.70	8	1.618	.144
MTF	5	13.65	1.89					
MK1V	5	17.21	2.03	1.12	6.59	8	3.252*	.012
MK1F	5	13.35	1.71					
MK2V	5	18.30	4.76	−0.97	11.63	8	1.950	.087
MK2F	5	12.97	3.83					
DTV	5	6.41	0.93	−1.13	2.02	8	0.657	.530
DTF	5	5.96	1.21					
DRV	5	8.20	0.67	−1.45	1.50	8	0.038	.971
DRF	5	8.17	1.27					
DK1V	5	5.45	0.88	−2.48	0.74	8	1.249	.247
DK1F	5	6.32	1.29					
DK2V	5	5.02	0.72	−2.76	−0.48	8	3.270*	.011
DK2F	5	6.63	0.84					
STV	5	8.59	0.88	−7.13	−4.29	8	9.275***	<.001
STF	5	14.30	1.06					
SRV	5	9.47	0.85	−3.46	−1.21	8	4.785**	.001
SRF	5	11.81	0.68					
SK1V	5	8.23	1.47	−7.43	−3.73	8	6.945***	<.001
SK1F	5	13.85	1.03					
SK2V	5	8.18	1.62	−7.48	0.93	8	8.069***	<.001
SK2F	5	15.66	1.30					

Abbreviations: D, disc; F, *Filtek*; K1, Khat 1; K2, Khat 2; M, Mylar; R, red wine; S, White stone; T, tea; V, *Vit‐l‐escence*.

* *p* < .05

** *p* < .01

*** *p* < .001.

For (M) finished samples, in (R), *Filtek* specimens were more color stable than *Vit‐l‐escence* specimens for the entire duration of staining (*p* = .001 for mean Δ*E* after 14 days). In (T), *Vit‐l‐escence* specimens had less mean Δ*E*, except for readings taken at 10 days (240 h). In both (K1) and (K2), *Filtek* specimens were more color stable during the staining period (*p* = .012 for mean Δ*E* after 14 days for K1).

For *Soflex* disc (D) finished samples, in (R) and (T), the two materials demonstrated comparable color difference. In (K1) and (K2), *Vit‐l‐escence* specimens had better color stability during the staining period (*p* = .011 for mean Δ*E* after 14 days for K2).

For White stone (S) finished samples, *Vit‐l‐escence* specimens demonstrated less mean Δ*E* during the staining period in all the staining solutions (*p* ≤ .001 for mean Δ*E* after 14 days).

**Figure 2:**
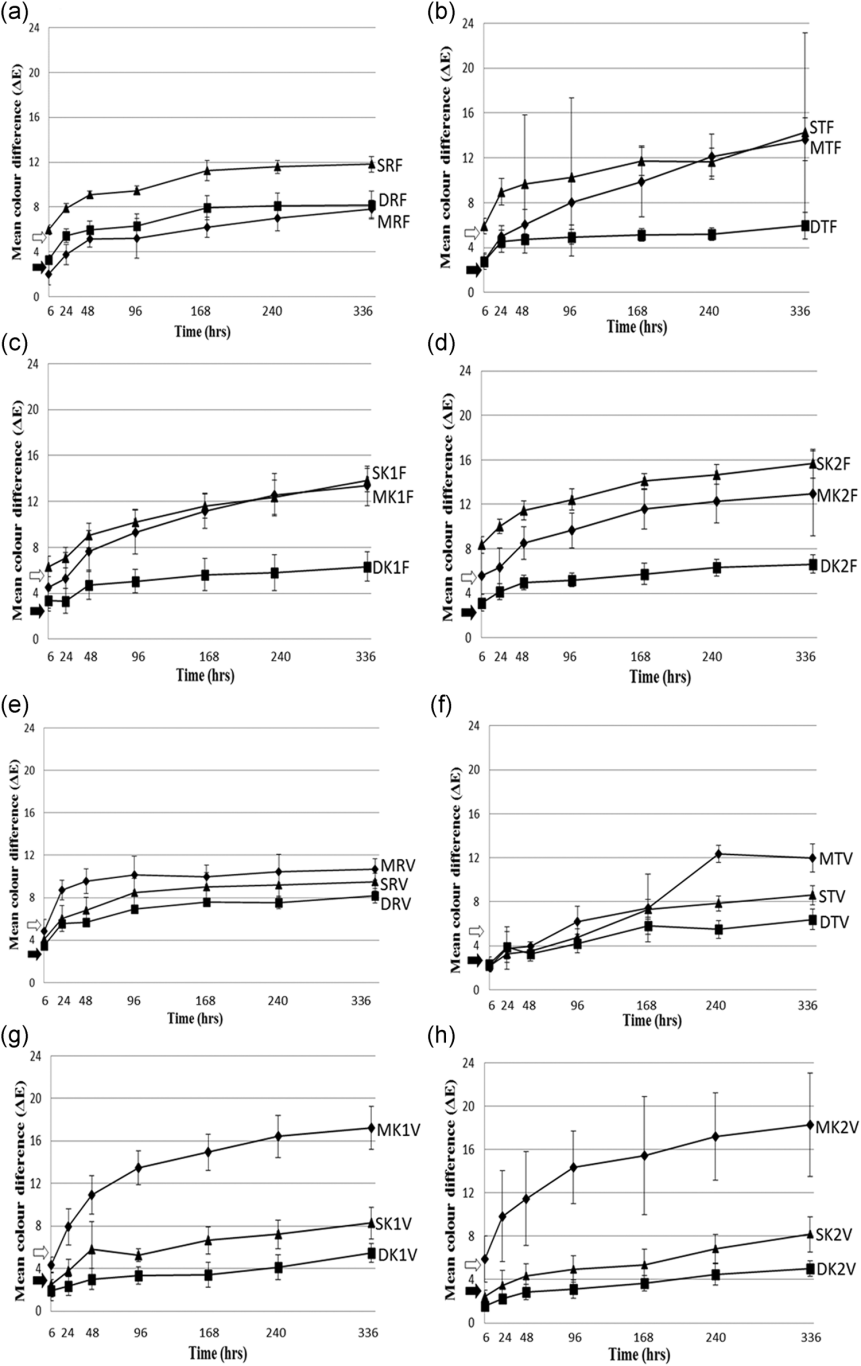
Mean color difference (Δ*E*) of *Filtek* (F) and *Vit‐l‐escence* (V) specimens finished by Mylar (M), Soflex disc (D), and White stone (S), and immersed in red wine (R)—(a) and (e); tea (T)—(b) and (f); Khat 1 (K1)—(c) and (g), and Khat 2 (K2)—(d) and (h). Black arrow, clinically perceptible Δ*E* level; white arrow, clinically unacceptable Δ*E* level (error bars denote SD)

Figure 2 compares the effect of the three finishing protocols on *Filtek* and *Vit‐l‐escence* samples in red wine (A, E), tea (B, F), Khat 1 (C, G), and Khat 2 (D, H). Table 3 shows the results of the independent‐sample *t* tests for the same. Table 4 shows that ANOVA test elicited a statistically significant difference in mean color (Δ*E*) between and within the specimens (*F* = 42.658, *p* < .001).

**Table 3:** Independent‐sample *t* tests of mean Δ*E* of M, *Soflex* D, and S finished V or F specimens in R, T, K1, and K2 at 14 days

Specimen		Mean difference	95% Confidence interval		*p*
			Lower bound	Upper bound	
MRF^case^	DRF^case^	−0.35	−1.95	1.25	.828
	SRF^case^	−3.99***	−5.59	−2.39	<.001
SRF^case^	MRF^case^	3.99***	2.39	5.59	<.001
	DRF^case^	3.63***	2.03	5.23	<.001
MRV^case^	DRV^case^	2.46**	1.01	3.91	.002
	SRV^case^	1.18	−0.27	2.63	.116
DRV^case^	MRV^case^	−2.46**	−3.91	−1.01	.002
	SRV^case^	−1.28	−2.72	0.17	.087
DTF^case^	MTF^case^	7.69	−1.19	16.57	.093
	STF^case^	−4.57	−13.45	4.30	.384
MTF^case^	DTF^case^	−7.69	−16.57	1.19	.093
	STF^case^	−12.26**	−21.14	−3.39	.008
DTV^case^	MTV^case^	5.60***	3.84	7.35	<.001
	STV^case^	3.41***	1.66	5.17	<.001
MTV^case^	DTV^case^	−5.60***	−7.35	−3.84	<.001
	STV^case^	−2.18*	−3.94	−0.43	.016
MK1F^case^	DK1F^case^	7.03***	4.72	9.34	<.001
	SK1F^case^	−0.50	−2.81	1.80	.833
DK1F^case^	MK1F^case^	−7.03***	−9.34	−4.72	<.001
	SK1F^case^	−7.53***	−9.84	−5.22	<.001
MK1V^case^	DK1V^case^	11.76***	9.17	14.35	<.001
	SK1V^case^	8.93***	6.34	11.52	<.001
DK1V^case^	MK1V^case^	−11.76***	−14.35	−9.17	<.001
	SK1V^case^	−2.83*	−5.42	−0.24	.032
MK2F^case^	DK2F^case^	6.34**	2.31	10.36	.003
	SK2F^case^	−2.69	−6.71	1.34	.217
DK2F^case^	MK2F^case^	−6.34**	−10.36	−2.31	.003
	SK2F^case^	−9.02***	−13.05	−5.00	<.001
MK2V^case^	DK2V^case^	13.28***	8.33	18.22	<.001
	SK2V^case^	10.12***	5.18	15.06	<.001
DK2V^case^	MK2V^case^	−13.28***	−18.22	−8.33	<.001
	SK2V^case^	−3.16	−8.10	1.79	.244

Abbreviations: D, disc; F, *Filtek*; K1, Khat 1; K2, Khat 2; M, Mylar; R, red wine; S, White stone; T, tea; V, *Vit‐l‐escence*.

* *p* < .05

** *p* < .01

*** *p* < .001.

**Table 4:** ANOVA comparing mean color difference of all samples

Source	Sum of squares	*df*	Mean square	*F*	*p*
Between groups	2008.678	29	69.265	42.658	<.001
Within groups	194.848	120	1.624		
Total	2203.526	149			

Abbreviation: ANOVA, analysis of variance.

For *Filtek*, (S) finished samples showed the highest mean Δ*E* in all staining solutions throughout the staining period; in (R), (M) finished samples recorded the least color difference, whereas in (T), (K1), and (K2), (D) finished samples recorded the least Δ*E* throughout the staining period. There were statistically significant differences between the various finishing protocols in the various staining solutions (*p* < .001).

For *Vit‐l‐escence*, in all the staining solutions, (M) finished samples showed the highest mean ΔE while (D) finished samples recorded the least throughout the staining period. There were statistically significant differences between the various finishing protocols in the various staining solutions (*p* < .001).

## 4 DISCUSSION

Resin composite restorations are exposed to diverse stains and chemo‐mechanical effects in the oral environment culminating in discolouration as demonstrated by the nanofill (*Filtek*) and microhybrid (*Vit‐l‐escence*) composite materials evaluated in this study. Both materials depicted unacceptable discoloration with a mean Δ*E* > 5.5 units after 14 days, except *Soflex* disc‐finished microhybrid specimens in khat. Based on the results, the null hypothesis was rejected. There was a significant difference in the color stability of nanofill and microhybrid dental resin composite restorative materials with different surface finishing treatments in different staining solutions.

In this study, the clinically perceptible Δ*E* was considered to be 2.6 units and the clinically unacceptable Δ*E* was 5.5 units, employing the previously determined color matching tolerances of dentists (Douglas et al., 2007). Distilled water was the control and did not produce a clinically perceptible Δ*E* in any of the specimens, which is consistent with observations from several other studies (Ertaş et al., 2006; Falkensammer et al., 2013; Guler et al., 2005), indicating that adsorption of water did not cause significant color change. The highest mean Δ*E* was observed in the Mylar‐finished microhybrid composite in the diluted khat (K2), whereas the lowest was observed in the same solution and sample with a *Soflex* disc finish. The SDs for color difference were generally high for mylar and low for stone‐ and disc‐finished specimens. This may be due to unpredictability of uniformity on the surface layers after mylar finish in comparison with the other two finishing protocols.

Overall, the disc finish resulted in the best color stability in both the microhybrid and the nanofill composites in all staining solutions over the duration of the study. It was only in red wine that the mylar finish resulted in less Δ*E* than the disc finish for the nanofill. Generally, the stone finish in tea and khat resulted in highest Δ*E* in the nanofill, whereas the mylar finish in khat resulted in the highest Δ*E* in the microhybrid. This is consistent with findings from other studies, which also reported better outcome with *Soflex* disc than mylar finish (Kumari et al., 2015; Schmitt et al., 2011).

Although surface roughness of the samples was not analyzed after finishing, the mylar‐finished specimens were expected to produce the least surface roughness. A smooth finish can improve esthetics and longevity and the smooth finish achieved with the mylar strip is regarded as the gold standard for composites (Catelan et al., 2013). This assumes that the superficial layer contains more polymer matrix than fillers (Kumari et al., 2015). Although this layer is advantageous for a smooth surface, it becomes a disadvantage when its effect on Δ*E* is considered, due to its high affinity for water and stains, and may explain the high Δ*E* observed in the (M) finished samples and particularly the microhybrid composites. However, this layer is removed during the disc and stone finishes (Schmitt et al., 2011). Moreover, the disc was expected to produce a smoother finish compared to the stone, as was observed in both materials. The disc system incorporates a multistep polishing technique utilizing sequential discs with decreasing abrasiveness, which results in a smoother final finish compared to single‐step materials such as stone (Schmitt et al., 2011; Turkun & Turkun, 2004). These findings are similar to those reported by several other investigators who have also associated mylar finish with poor color stability (Berber et al., 2013; Patel et al., 2004; Yew et al., 2013).

As the stone finish eliminates the resin‐rich superficial layer, it was expected to result in better color stability than mylar. This was the case with the microhybrid but not the nanofill resin composite. As stated by the manufacturer, *Filtek* contains 63.3%, whereas *Vit‐l‐escence* contains 52% filler content by volume. Higher filler content translates to lower resin content in the superficial layer; therefore, the stone on a slow‐speed motor may not be an effective finishing protocol for such composites with higher filler content as the nanofills.

By the end of the staining period, all stone‐ and mylar‐finished samples for both materials demonstrated statistically significant Δ*E* above the clinically unacceptable tolerance. For the disc finish in tea and red wine, there was not statistically significant Δ*E* between the two materials. Red wine is known to cause significant Δ*E* in composites (Ertaş et al., 2006; Falkensammer et al., 2013; Kisumbi, 1998; Silva et al., 2014) and it has been proposed that alcohol causes softening of the resin matrix of the composite facilitating staining (Topcu et al., 2009). Several studies have also reported that tea can cause significant discoloration of dental composites due to adsorption of colorants in the form of tannins (Ghahramanloo, 2008; Malekipour et al., 2012; Omata et al., 2006). Staining of teeth from khat chewing has been attributed to direct staining by chemicals (tannins) as well as khat‐favoring chromogenic bacteria (Aden et al., 2006; Al‐Maweri et al., 2018; Astatkie et al., 2015); likewise, it is assumed that the staining of specimens in this study was caused by the tannins in khat. In both khat solutions, the nanofill composite demonstrated less Δ*E* for the mylar finish, whereas the microhybrid demonstrated less Δ*E* with the disc and stone finishes. Interestingly, diluted khat resulted in higher Δ*E*, hence greater staining, in stone‐finished *Filtek* specimens and mylar‐finished *Vit‐l‐escence* specimens compared with undiluted khat. This suggests that dilution avails more khat extract, increasing its propensity to cause staining in certain situations. In stone‐finished samples for both materials, the Δ*E* in the two khat solutions was comparable. As was previously explained, it is apparent that stone finish results in decreased color stability of the nanofill, whereas the mylar finish results in decreased color stability of the microhybrid composites.

## 5 CONCLUSION AND CLINICAL SIGNIFICANCE

The findings of this study may not be directly applied to the clinical situation because of the continuous staining procedure, absence of the effects of the complex oral environment, and habitual oral hygiene effects; nonetheless, they provide an indication of the effect of these staining solutions on the color stability of dental resin composites. Within the limitations of this study, we concluded that, overall, *Soflex* discs finish resulted in better color stability compared to White polishing stone and Mylar finish for both the nanofill and the microhybrid composites; therefore, clinicians may accomplish best results if they complete these restorations using the *Soflex* discs. All staining solutions caused clinically unacceptable discolouration of all specimens finished with mylar and white stone. For *Soflex* finish, red wine produced clinically unacceptable color difference beyond 48 h; therefore, the effect of the staining solution was found to be dependent on the polishing protocol, which ideally should be determined by the type of filler system. Patients should be advised to consume potentially staining foods or beverages in moderation.

## CONFLICTS OF INTEREST

The authors declare no conflicts of interest.

## AUTHOR CONTRIBUTIONS

Chamunorwa Marufu, Bernina K. Kisumbi, Olivia A. Osiro, and Fred O. Otieno contributed to conception and design, contributed to acquisition, analysis, and interpretation of data, and drafted and critically revised the manuscript. All authors gave their final approval and agree to be accountable for all aspects of the work.

## Data Availability

The data that support the findings of this study are available from the corresponding author upon reasonable request.
